# Palliative Care for Cancer Patients During the COVID-19 Pandemic, With Special Focus on Lung Cancer

**DOI:** 10.3389/fonc.2020.01405

**Published:** 2020-07-21

**Authors:** Janna J. A. O. Schoenmaekers, Lizza E. L. Hendriks, Marieke H. J. van den Beuken-van Everdingen

**Affiliations:** ^1^Department of Pulmonary Diseases, GROW-School for Oncology and Developmental Biology, Maastricht University Medical Centre+, Maastricht, Netherlands; ^2^Centre of Expertise for Palliative Care, Maastricht University Medical Centre+, Maastricht, Netherlands; ^3^Department of Optimising Patient Care, School for Care Public Health Research Institute (CAPHRI), Maastricht University, Maastricht, Netherlands

**Keywords:** coronavirus, pandemic, lung cancer, palliative care, telemedicine

## Introduction

The COVID-19 pandemic causes a massive shift in the distribution of health care. Due to the high number of patients needing hospitalization and even intensive care unit (ICU) treatment, health care professionals (HCP) are often redeployed from their original specialization to COVID-19 treatment. This potentially compromises the care for others, such as oncology patients. Furthermore, cancer patients, the elderly, and those with comorbidities are at high risk of developing COVID-19 related morbidity and mortality (1, 2). This is especially true for lung cancer patients, with reported mortality rates of 25–55% (3–6), compared with up to 10% in other COVID-19 patients (5),which can be explained by the fact that several risk factors for severe COVID-19 can be found in the group of patients with lung cancer: cancer itself, its potentially immunosuppressive treatment, a high median age (>70 years), often a smoking history, and a high percentage of comorbidities (7–10). Furthermore, outpatient clinic, daycare as well as hospital visits might increase the risk of contracting COVID-19. To minimize COVID-19 related risks, the risk/benefit ratio for all (lung) cancer treatments, including systemic anticancer treatment must be reconsidered (11). During infectious disease outbreaks, actions taken by public health authorities to limit the spread of disease can exacerbate psychological, social, and spiritual suffering, as was seen during the Ebola epidemic in 2014-2015 and the SARS epidemic in 2002 (12, 13). However, already in 2018, the World Health Organization (WHO) stated: “In epidemics of life-threatening infections, as in other Humanitarian emergencies and crises, the suffering of victims and efforts to relieve it, often are neglected in haste to save lives. Yet, palliative care in these situations is just as critical” (14).

Till now, for (lung) cancer, the focus has been on how to optimize treatment pathways during the pandemic. The continuation of palliative care is essential as well since early integration of palliative care leads to significant improvements in quality of life (QoL), mood, and possibly also longer overall survival (OS) (15). However, during the COVID-19 pandemic, palliative care isjeopardized in all four pillars (physical, psychological, social, and spiritual). Patients are anxious to visit the hospital and HCP are encouraged to avoid face-to-face contacts as much as possible (16).

As the first wave of the COVID-19 pandemic seems to be behind us in Europe, and the health care systems return to normal, we can look back and reflect on things we have learned until now. We can also use this knowledge in countries where the pandemic is still ongoing. In this opinion article, we will focus on the impact that COVID-19 had on palliative care for (lung) cancer patients, and we will provide practical advice on how to maintain and improve palliative care in times of a resurgence of the virus. A summary is also provided in Table 1.

**Table 1:** Summary problems and possible solutions of palliative care for non-COVID lung cancer patients during the COVID-19 pandemic.

**Domain of palliative care**	**Problems during COVID-19 pandemic**	**Possible solution**
Psychological	- Anxiety for COVID-19 but also for cancer treatment changes - Depression due to social isolation	- Self-care and distress management tools. Links with examples of self-care tools for patients or clinicians are found below. • https://www.nccn.org/covid-19/pdf/NCCN_Patient_Self_Care_COVID-19.pdf (17) • https://helixcentre.com/project-end-of-life-toolkit (18) • https://www.nccn.org/covid-19/pdf/Distress-Management-Clinician-COVID-19.pdf (19) - Telemedicine
Social	- Social distancing and therefore lack of social support	- Technical applications for video calling
Physical	- Shortage of drugs - Shortage of materials such as pump drivers - Shortage of caregivers - Minimized face-to-face contact between treating physician and patient	- Adapting guidelines with alternative drugs and pump systems. If a shortage is impending it is recommended to look at the local (changed) guidelines, because the local availability of products can be different in every region. Links to currently adapted guidelines are found below. • https://www.palliativecareguidelines.scot.nhs.uk/guidelines/symptom-control/alternatives-to-regular-medication-normally-given-via-a-syringe-pump-when-this-is-not-available.aspx (20) • https://www.esmo.org/content/download/286441/5690599/1 (21) • https://www.esmo.org/content/download/286432/5690545/1 (22) - Telemedicine - Adapting palliative care for specific complaints • Painful bone metastases: avoid RTx when pain controlled by level 1–3 oral analgesics. If RTx necessary, mono-fractionated (8 Gy in 1 fr) (23) • Thoracic RTx: palliative RTx for symptomatic mediastinal/hilar mass → not postpone treatment but change to a more hypofractionated regimen (24) • Malignant pleural effusions: pleural indwelling catheter when in need of frequent pleural fluid drainage (25)
Spiritual	- Loss of sense of meaning of life - Loss of faith	- Seek partnership with local spiritual counselors

*COVID-19, corona virus disease 2019; RTx, radiotherapy; Gy, Gray; fr, fraction*.

## Palliative Care During COVID-19

### Palliative Care Services and Redeployment of Personnel

Palliative care teams and hospice services have an essential role in response to COVID-19. A systematic review identified the following important issues: a rapid and flexible response, availability of protocols for symptom management, palliative care specialists involved in triage, the possibility of shifting resources from inpatient to community settings (the demand there may be higher), the possibility of redeploying volunteers to provide psychosocial, and bereavement care and the use of technology to communicate with patients and caregivers (26). This is described in more detail below.

The workload for palliative care teams increased during the pandemic. Because most teams are small in number and the different providers have complementary roles, it is vital to keep the existing palliative care providers free from COVID-19 (27). By coaching primary health care teams with guidelines and order sets for the management of COVID-19 symptoms, the optimal use of palliative care consultation for non-COVID-19 patients could be ensured.

Caregivers and volunteers in hospice care were at higher risk of and had a fear of infection; as a result, personnel and volunteers fell short. Therefore, some hospices even closed their doors (28, 29). The shortage of home-care nurses due to an increase in patients and illness of the nurses themselves was a threat to the delivery of adequate palliative care. There was even set up an agreement that in case of lack of community nurses to administer drugs, a family member could be taught, under strict conditions, to deliver intermittent morphine subcutaneously, although this was not desirable. This could save HCP as the syringes can be prepared for 24 h of drug administration, once daily, by trained nurses.

### Palliative Care Services and Scarcity of Resources

Besides the understaffing, other resources ran short (such as face masks and other protective equipment). There was a (risk of) shortages in certain commonly used drugs such as morphine, midazolam, lorazepam, and propofol. These are all medications used to relieve symptoms in the palliative and terminal stages of patients with cancer. Morphine has been proven to be an effective treatment for refractory breathlessness (30, 31). The risk of a shortage of morphine existed, but other drugs are not effective [oxycodone (32)], or only anecdotal proof of its effectiveness on dyspnea exists [fentanyl (33, 34)]. To guide physicians in the use of other opioids in the treatment of dyspnea, local guidelines rapidly changed, or expanded with recommendations in times of shortages. For example, in the Netherlands, the guideline “symptomatic treatment of dyspnea” temporarily expanded with recommendations concerning the use of Rapid Onset Opioids (ROO's). Importantly, these recommendations have also been adopted by the European Society for Medical Oncology (ESMO) (21, 22).

Midazolam and propofol, delivered as a continuous intravenous or subcutaneous infusion, are used in protocols for palliative sedation. The impending shortages of medication and pumps asked for “what if” scenarios. As an example, in the Netherlands, a new guideline “palliative sedation in times of COVID-19” was written by a group of palliative care experts and endorsed by the relevant scientific associations, including ESMO (21, 22).

Other countries, such as the United Kingdom, have also rapidly set up guidelines on how to act when conventional drugs and pump systems are not available. Therefore, when a shortage in certain drugs is foreseen in a second COVID-19 wave, it is recommended to look at the local (changed) guidelines, because the local availability of products can be different in every region (20). The links to the currently available guidelines can be found in Table 1.

### Telemedicine

Flexibility and resilience have been fundamental tools to overcome critical issues. The health care system adapted fast to the changing practice and telemedicine was quickly implemented. Previously, the use of telemedicine in palliative care showed improvements in symptom management, comfort with care, and patient and family satisfaction (35). It also facilitated cooperation between the community nurses and the specialized palliative care team nurse (36). Still, potential barriers were technology-related complications in the elderly and frail population (35) and private issues regarding the illness, with family members present (36). Moreover, patients, as well as physicians, did not always feel comfortable with non-face-to-face physician care. Telemedicine also has hidden advantages: it allows HCP to see patients' home environments, information that is usually lacking. Before considering telemedicine the norm, it is important to remember that at least one randomized controlled trial (RCT) of weekly palliative care teleconsultations vs. in-person palliative care showed significantly more anxiety and higher distress in the telehealth group (37, 38). The corona crisis forced patients, their families, and HCP to implement telemedicine rapidly. This was also for the inpatients due to quarantine with increasingly isolated hospitalized patients (39). However, videoconferencing and online services stay second- best in palliative care. It cannot replace the sitting-down with a patient, nor the comforting touch, but it enabled HCP to pick-up non-verbal clues and emotions essential for good communication.

## Changes Due to COVID-19 Pandemic in Four Pillars of Palliative Care

### Physical

Palliative care for specific complaints has been adapted as well. The ESMO published guidance for cancer care during COVID-19, including a part on palliative care prioritization. The underlying principles included: (1) continuing to prioritize the relief of severe distress and the management of severe acute complications of cancer (such as cord compression, symptomatic brain metastases in patients with good performance status, pleural effusion, severe dyspnea, pain, etc.), (2) managing many symptom control issues by telephone when possible, and (3) arranging home care services whenever possible for patients with anticipated high palliative care needs (40). To minimize the risk for patients, time they spend at the hospital should be reduced. Recommendations for specific complaints and treatments such as radiotherapy are described in Table 1, and will not be described in detail in the text.

### Psychological and Social

For (lung) cancer patients, the psychosocial impact of the COVID-19 pandemic was substantial. During the peak, social life was disrupted because of social distancing. Visits from friends and family were lacking, but also the visits of formal /informal caregivers were limited. Due to the fear of becoming infected, patients isolated themselves even more (41). However, social support has been shown to improve QoL in lung cancer patients (42), and lung cancer patients indicate that they indeed need family support during treatment (43). In colorectal cancer, good social integration was even associated with improved survival (44).

Besides anxiety and fear due to the diagnosis of cancer, in the COVID-19 pandemic, these complaints also arose from adjusted or postponed treatments. Therefore, uncertainty about the future increased. Also, the duration of the pandemic has been uncertain, and unfortunately, straightforward information regarding the lung cancer treatment plan was lacking. In patients treated with palliative intent, outside of the COVID-19 pandemic, fear of metastases and insecurity about the future is already increased compared with those treated with curative intent (43). The Dutch federation of cancer patient organizations (NFK) collected through a survey the experiences of patients with cancer. Twelve percent of the responders were lung cancer patients. Fifty-five percent of all patients were worried to contract a COVID-19 infection, and 26% had (several) concerns regarding the consequences of COVID-19 on anti-cancer treatment or follow-up (45). Palliative care in this patient population is important, as it has been previously shown that this can reduce fear and anxiety (46). The National Comprehensive Cancer Network (NCCN) has developed a self-care and distress management tool for cancer patients, with information about how to obtain up-to-date information, manage distress and improve resilience, but this tool does not specifically focus on patients treated with palliative intent (17).

Self-isolation affects not only the lung cancer patient but also their informal caregivers, as social support and psychosocial interventions, although effective (47), decreased for this group, while the burden of caregiving remained. Furthermore, informal caregivers were increasingly confronted with patients dying at home, as hospices were less available, and professional HCP reduced their visits. To anticipate problems and questions, end-of-life toolkits were developed, for example, in the United Kingdom (18).

COVID-19 related mortality is higher in cancer patients compared with the general patients. An international registry-based cohort study showed a mortality rate of 33% among patients with thoracic cancer (6). As “normal” mourning and burial rituals were not possible due to the social distancing, the mourning of loved ones that stay behind will be influenced (48).

Visits to the outpatient clinic were reduced in most hospitals, and most of the consultations were by telephone. Not all hospitals had videoconferencing in place. Therefore, non-verbal communication was hampered. Without non-verbal communication, psychological stress from patients or relatives is less recognized (49), and empathy and respect toward the patient are less conveyed (50).

Importantly, non-verbal communication can help recall information given in the consultation by the patient (51), which would improve therapy adherence and probably also reduce insecurity, fear, and anxiety in patients, and their relatives.

Furthermore, due to shifting tasks and a higher number of working hours, combined with the risk of COVID-19 infection, HCP themselves are at risk of psychosocial problems or burnout (52). This also limited their ability to provide palliative care for patients (53). Organizations such as the NCCN have also published toolkits for HCP (19).

### Spiritual

The importance of spirituality in coping with uncertainty, severe disease, and at the end of life is recognized. Spiritual well-being offers some protection against end-of-life despair in those for whom death is imminent (54). There is a positive and significant relationship between spiritual well-being, mental health, and QoL in cancer patients (55). Spiritual suffering during the COVID-19 pandemic can intensify the feeling of loss of sense of the meaning of life and even loss of faith. Therefore, it is important to seek partnership with local spiritual counselors willing to visit patients and family members on request face-to-face when possible or otherwise with telemedicine (14).

## Conclusion

The COVID-19 pandemic changed the health care systems. As face-to-face contacts were severely reduced, and there was a risk of a shortage of specific resources used in palliative care medicine, palliative care for (lung) cancer patients was jeopardized.

By changing and evolving current guidelines rapidly and adopting new ways of communication with, for instance, telemedicine palliative care could be maintained as good as possible.

An important lesson to be learned from this crisis is that resilience and flexibility of the health care system and HCP are crucial.

## Author Contributions

All authors listed have made a substantial, direct and intellectual contribution to the work, and approved it for publication.

## Conflict of Interest

The authors declare that the research was conducted in the absence of any commercial or financial relationships that could be construed as a potential conflict of interest.
